# The impact of the pathogen *Rhizoctonia solani* and its beneficial counterpart *Bacillus amyloliquefaciens* on the indigenous lettuce microbiome

**DOI:** 10.3389/fmicb.2014.00175

**Published:** 2014-04-21

**Authors:** Armin Erlacher, Massimiliano Cardinale, Rita Grosch, Martin Grube, Gabriele Berg

**Affiliations:** ^1^Institute of Environmental Biotechnology, Graz University of TechnologyGraz, Austria; ^2^Institute of Plant Sciences, University of GrazGraz, Austria; ^3^Leibniz-Institute of Vegetable and Ornamental CropsGrossbeeren, Germany

**Keywords:** lettuce microbiome, Gammaproteobacteria, soil-borne pathogens, 16S rRNA gene pyrosequencing, phyllosphere, rhizosphere, Lactuca sativa

## Abstract

Lettuce belongs to the most commonly raw eaten food worldwide and its microbiome plays an important role for both human and plant health. Yet, little is known about the impact of potentially occurring pathogens and beneficial inoculants of the indigenous microorganisms associated with lettuce. To address this question we studied the impact of the phytopathogenic fungus *Rhizoctonia solani* and the biological control agent *Bacillus amyloliquefaciens* FZB42 on the indigenous rhizosphere and phyllosphere community of greenhouse-grown lettuce at two plant stages. The rhizosphere and phyllosphere gammaproteobacterial microbiomes of lettuce plants showed clear differences in their overall and core microbiome composition as well as in corresponding diversity indices. The rhizosphere was dominated by Xanthomonadaceae (48%) and Pseudomonadaceae (37%) with *Rhodanobacter, Pseudoxanthomonas, Dokdonella, Luteimonas, Steroidobacter, Thermomonas* as core inhabitants, while the dominating taxa associated to phyllosphere were Pseudomonadaceae (54%), Moraxellaceae (16%) and Enterobacteriaceae (25%) with *Alkanindiges*, *Pantoea* and a group of Enterobacteriaceae unclassified at genus level. The preferential occurrence of enterics in the phyllosphere was the most significant difference between both habitats. Additional enhancement of enterics on the phyllosphere was observed in bottom rot diseased lettuce plants, while *Acinetobacter* and *Alkanindiges* were identified as indicators of healthy plants. Interestingly, the microbial diversity was enhanced by treatment with both the pathogen, and the co-inoculated biological control agent. The highest impact and bacterial diversity was found by *Rhizoctonia* inoculation, but FZB42 lowered the impact of *Rhizoctonia* on the microbiome. This study shows that the indigenous microbiome shifts as a consequence to pathogen attack but FZB42 can compensate these effects, which supports their role as biocontrol agent and suggests a novel mode of action.

## Introduction

Plants host a broad range of ubiquitous but also highly adapted and specific bacterial communities that colonize their epi- and endophytic compartments (Berg and Smalla, 2009; Berendsen et al., 2012; Bulgarelli et al., 2012). Due to their complexity, specific morphology, and production of secondary metabolites, the structure and function of plant-associated microbial communities are specific in these habitats but also at plant species and cultivar levels (Smalla et al., 2001; Berg et al., 2002; Berg and Smalla, 2009; Raaijmakers et al., 2009). The rhizosphere has already been investigated as a microbial habitat for more than one century (Hartmann et al., 2008), while the phyllosphere microbiome is only partly understood. Recent work suggests that the long-term colonization of phyllosphere is preferred by specific bacteria, while short-time colonization comprises many ubiquitous bacteria (Vorholt, 2012). All plant-associated habitats contain a high proportion of plant-beneficial microorganisms such as antagonists, diazotrophs, and plant growth promoting bacteria (PGPB) but also plant pathogens as well as potential human pathogens (Berg et al., 2005; Mendes et al., 2013). While the modes of action are often understood for single beneficial as well as pathogenic strains and species, less is known about the microbial community impact of single strains. Risk assessment and colonization studies for specific biocontrol agents showed minor and only transient effects on the rhizosphere community (Scherwinski et al., 2007; Adesina et al., 2009; Chowdhury et al., 2013; Schmidt et al., 2014), while impacts of pathogens on the indigenous microbiome are severely underexplored.

Soil-borne plant pathogens cause crucial damage to crops. The phytopathogenic fungus *Rhizoctonia solani* Kühn [teleomorph: *Thanatephorus cucumeris* (A.B. Frank) Donk; basidiomycetes] is subdivided into anastomosis groups (AGs) according to their hyphal anastomosis reactions (Carling et al., 2002). The fungus causes a wide range of commercially significant plant diseases, such as Brown patch, damping off in seedlings, root rot and belly rot. *R. solani* strains are characterized by a distinct degree of host specificity as well as by different virulence levels to their plant host. *Rhizoctonia* strains occur almost ubiquitously in soils but isolates AG1-IB (Germany), AG2-1 (UK, the Netherlands) and AG4 (the Netherlands, UK, USA) have been isolated from diseased lettuce plants (Grosch et al., 2004). Strains belonging to AG1-IB were responsible for up to 70% yield loss of field-grown lettuce (Davis et al., 1997; Wolf and Verreet, 1999). One strain 7/3/14 of the supposed diploid and heterokaryotic *R. solani* AG1-IB, which was already sequenced, shows a large genome with many unique and unknown features in comparison with other *Rhizoctonia* strains and phylogenetically related fungi (Wibberg et al., 2013). Due to the low degree of host specificity, *Rhizoctonia* strains of different AGs can lower the general fitness of the plant during colonization, which results in higher sensitivity for additional pathogens such as spoilage enterobacteria (Berg et al., 2005). Interestingly, Adesina et al. (2009) could demonstrate direct changes caused by *R. solani* to the fungal and bacterial community patterns using molecular fingerprinting. All *Rhizoctonia* diseases, and subsequent secondary infections, in plants are difficult to control. In the past, only methyl bromide (MeBr) was effectively used. However, this fumigant has been banned for its ozone-depleting and toxic effects (UNEP, 1999). Alternative and environmentally friendly methods to suppress *Rhizoctonia* comprise naturally occurring antagonists such as *Serratia plymuthica* and *Pseudomonas jessenii* (Faltin et al., 2004; Grosch et al., 2005; Scherwinski et al., 2007; Adesina et al., 2009). In addition, *Bacillus amyloliquefaciens* FZB42, a long-time established plant strengthener was successfully applied to suppress *R. solani* on lettuce (Chowdhury et al., 2013). Genome sequencing of FZB42 revealed a high capacity of metabolite production with antimicrobial and antifungal activity, which suggested direct antifungal effects (Chowdhury et al., 2013). However, some reports suggest additional impacts of individual strains on the microbial community (Scherwinski et al., 2007; Schmidt et al., 2012). We therefore hypothesize that both beneficials as well as pathogens can cause significant shifts in the plant-associated microbiome.

The aim of this study was to identify the impact of the phytopathogenic fungus *R. solani* and the biological control agent *B. amyloliquefaciens* FZB42 on the indigenous rhizosphere and phyllosphere community of lettuce, cultivated under controlled conditions in a growth chamber. In our study we focus on the human health relevant group of Gammaproteobacteria, which was studied by analyzing specific amplicon libraries together with corresponding bioinformatic and statistical analysis. Gammaproteobacteria belong to the plant microbiome in general (Brandl, 2006), and are especially a substantial fraction of the lettuce-associated microbiome (Rastogi et al., 2012, 2013). However, they also comprise several species which were frequently identified to cause severe foodborne outbreaks (Teplitski et al., 2011).

## Material and methods

### Inoculants used in this study

The effect of *R. solani* and *B. amyloliquefaciens* FZB42 on lettuce growth and health was evaluated in this study. All experiments were performed with the product Rhizovital® 42 liquid (ABiTEP GmbH, Berlin, Germany), which is based on vital spores of FZB42 (Chowdhury et al., 2013). The bottom rot pathogen *R. solani* AG1-IB (isolate 7/3) was obtained from the strain collection of the Leibniz Institute of Vegetable and Ornamental Crops (Großbeeren, Germany) (Grosch et al., 2004).

### Experimental design of pot experiments

The effect of FZB42 and the pathogen *R. solani* on the microbial community of lettuce was studied by 454-amplicon sequencing analysis. Seeds (cv. Tizian, Syngenta, Bad Salzuflen, Germany) were germinated at 18°C in a seedling tray (92 holes) filled with a non-sterile mixture of quartz sand and substrate [Fruhstorfer Einheitserde Typ P, Vechta, Germany; chemical analysis (mg per l): N = 120, P = 120, K = 170, Mg = 120, S = 100, KCl = 1, organic substance = 167, peat = 309; pH 5.9] at a 1:1 ratio (v/v). The seedlings were further cultivated at 20/15°C until planting in a growth chamber (York, Mannheim, Germany; 16 h/8 h day/night cycle, 500 μmol m^−2^ s^−1^, 60/80% relative humidity). Lettuce was planted at two-leaf stage into pots (500 ml) filled with the same substrate sand mixture as mentioned above inoculated and non-inoculated with the pathogen *R. solani* AG1-IB and grown at 22/15°C for 4 weeks. In the treatments with pathogen inoculation, the substrate mixture was inoculated with 10 *R. solani*-infested barley kernels and incubated at 25°C for 1 week until planting of lettuce into the pots.

For application of the inoculant FZB42 each lettuce plant was drenched with 20 ml spore solution (10^7^ spores ml^−1^) 3 days before and at planting time respectively. The pots were watered lightly each day to maintain the substrate moisture, and fertilized weekly (0.2% Wuxal TOP N, Wilhelm Haug GmbH & Co. KG, Düsseldorf, Germany). All pot experiments were done at the Leibniz Institute of Vegetable and Ornamental Crops.

An overview about the sampling design is presented in Table 1. Here, abbreviations for compartments and treatments used throughout the manuscript were explained: P, phyllosphere; R, rhizosphere; Y, young; M, mature; RS, *R. solani*; C, untreated (control); FZB42RS, FZB42 and *R. solani* co-inoculation; G, healthy; K, diseased.

**Table 1:** **Sample design description**.

**ID**	**Habitat**	**Age (weeks after planting)**	**Treatment**	**Disease condition**	**Plant replicates**	**PCR replicates^b^**	**Sequencing**
PYC	Phyllosphere	Young (2)	Untreated (Control)	not determinable	2	2	MWG, Eurofins
PYRS	Phyllosphere	Young (2)	*R. solani* inoculated	not determinable	2	2	MWG, Eurofins
PYfzb42RS	Phyllosphere	Young (2)	*R. solani* and FZB42 inoculated	not determinable	2	2	MWG, Eurofins
PMG	Phyllosphere	Mature (4)	Untreated (Control)	healthy	3 (6^a^)	2	MWG, Eurofins
PMK	Phyllosphere	Mature (4)	*R. solani* and FZB42 inoculated	bottom rot	3 (6^a^)	2	MWG, Eurofins
RYC	Rhizosphere	Young (2)	Untreated (Control)	not determinable	2	2	Macrogen, Korea
RYRS	Rhizosphere	Young (2)	*R. solani* inoculated	not determinable	2	2	Macrogen, Korea
RYfzb42RS	Rhizosphere	Young (2)	*R. solani* and FZB42 inoculated	not determinable	2	2	Macrogen, Korea
RMG	Rhizosphere	Mature (4)	Untreated (Control)	healthy	3 (6^a^)	2	Macrogen, Korea
RMK	Rhizosphere	Mature (4)	*R. solani* and FZB42 inoculated	bottom rot	3 (6^a^)	2	Macrogen, Korea

a *Two Independent DNA extractions per plant were pooled prior to PCR*.

b *PCR was carried out twice for each DNA sample and each PCR step and pooled prior to sequencing*.

### Sample collection and DNA isolation

Sampling was carried out 2 weeks after planting (young plants) for treatments with and without FZB42 application, followed by a second sampling 4 weeks after planting (mature plants) for control and co-inoculated treatments with FZB42 and *R. solani*. The total community DNA was extracted per treatment, and habitat from two young plants and three mature plants (two independent DNA extractions were performed for each plant and the DNA was pooled prior to PCR), according to Bragina et al. (2011). Briefly, 5 g of plant material were physically disrupted with sterile pestle and mortar and resuspended in 10 ml of 0.85% NaCl. Two ml of suspension were centrifuged (16,500 × *g*, 20 min, 4°C) and the obtained pellets were used for isolation of the total-community DNA with the FastDNA® SPIN Kit for Soil (MP Biomedicals, Solon, OH, USA). For mechanical lysis, the cells were homogenized twice in a FastPrep® FP120 Instrument (Qbiogene, BIO101, Carlsbad, CA, USA) for 30 s at a speed of 5.0 m s^−1^ and treated according to the manufacturer's protocol.

### Barcoded deep 454-pyrosequening of 16S rRNA gene amplicon

The 16S rRNA genes of 24 samples (details are provided in Table 1) were amplified (two technical replicates for each sample) in a nested PCR approach with the Gammaproteobacteria primer set 395f (5′-CMA TGC CGC GTG TGT GAA-3′) and 871r (5′-ACT CCC CAG GCG GTC DAC TTA-3′) (Mühling et al., 2008). The PCR reaction mixture (20 μ l) contained 5 × Taq-&GO Ready-to-use PCR Mix (MP Biomedicals, Germany), 0.25 μM of each primer, 25 mM MgCl_2_ and 1 μl of template DNA (96°C, 4 min; 32 cycles of 96°C, 1 min; 57°C, 1 min; 74°C, 1 min; and final elongation at 74°C, 10 min). In a second PCR, 1 μl of the amplicon (1:10 diluted phyllosphere and 1:100 diluted rhizosphere derived PCR products) was used. 16S rRNA gene sequences were amplified by using the forward primer Unibac-II-515f (5′-GTG CCA GCA GCC GC-3′) containing the 454-pyrosequencing adaptors and the reverse primer Gamma871r_454 (5′-CTA TGC GCC TTG CCA GCC CGC TCA GAC TCC CCA GGC GGT CDA CTT A-3′). The reaction mixture for the second PCR (30 μ l) contained 5 × Taq-&GO Ready-to-use PCR Mix, 0.25 μ M of each primer (96°C, 4 min; 32 cycles of 96°C, 1 min; 66°C, 1 min; 74°C, 1 min; and final elongation at 74°C, 10 min). PCR products were purified using the Wizard SV Gel and PCR Clean-Up System (Promega, Madison, USA). The technical replicates per sample were pooled and the partial 16S rRNA gene fragments were sequenced using 454 Roche GS FLX (MWG Eurofins, Germany) and 454 Roche GS FLX Titanium (Macrogen Korea, South Korea) pyrosequencer. The nucleotide sequences obtained in this work were submitted to the European Nucleotide Archive (www.ebi.ac.uk/ena) and are available under the accession number PRJEB6022.

### DNA sequence analysis and taxonomical identification

Sequences were analyzed with the Qiime software version 6.0 (Caporaso et al., 2010). Replicates from sequencing of each treatment and habitat were bioinformatically pooled during the Qiime analysis for data evaluation. MID-, primer and adapter sequences were removed, length filtered (≥350 nt), quality filtered (score: 50), denoised, chloroplast removed and singletons adjusted. The cut-off level was set to 97% sequence identity. Chimeras were detected with Chimera Slayer and then removed. To compute alpha and beta diversity, the dataset was normalized to 5920 reads per sample. Ring-charts were created using the Krona software package version 2.2 (Ondov et al., 2011) and the profile network was constructed using Cytoscape version 3.0.2 (Shannon et al., 2003). Statistical tests based on the operational taxonomic units (OTUs) table were performed with the nonparametric ANOVA Kruskal Wallis test. This test is functionally an expansion of ANOVA to cases where the sample means are unequal and the distribution is not normal.

## Results

### The gammaproteobacterial microbiome of lettuce

**Figure 1:**
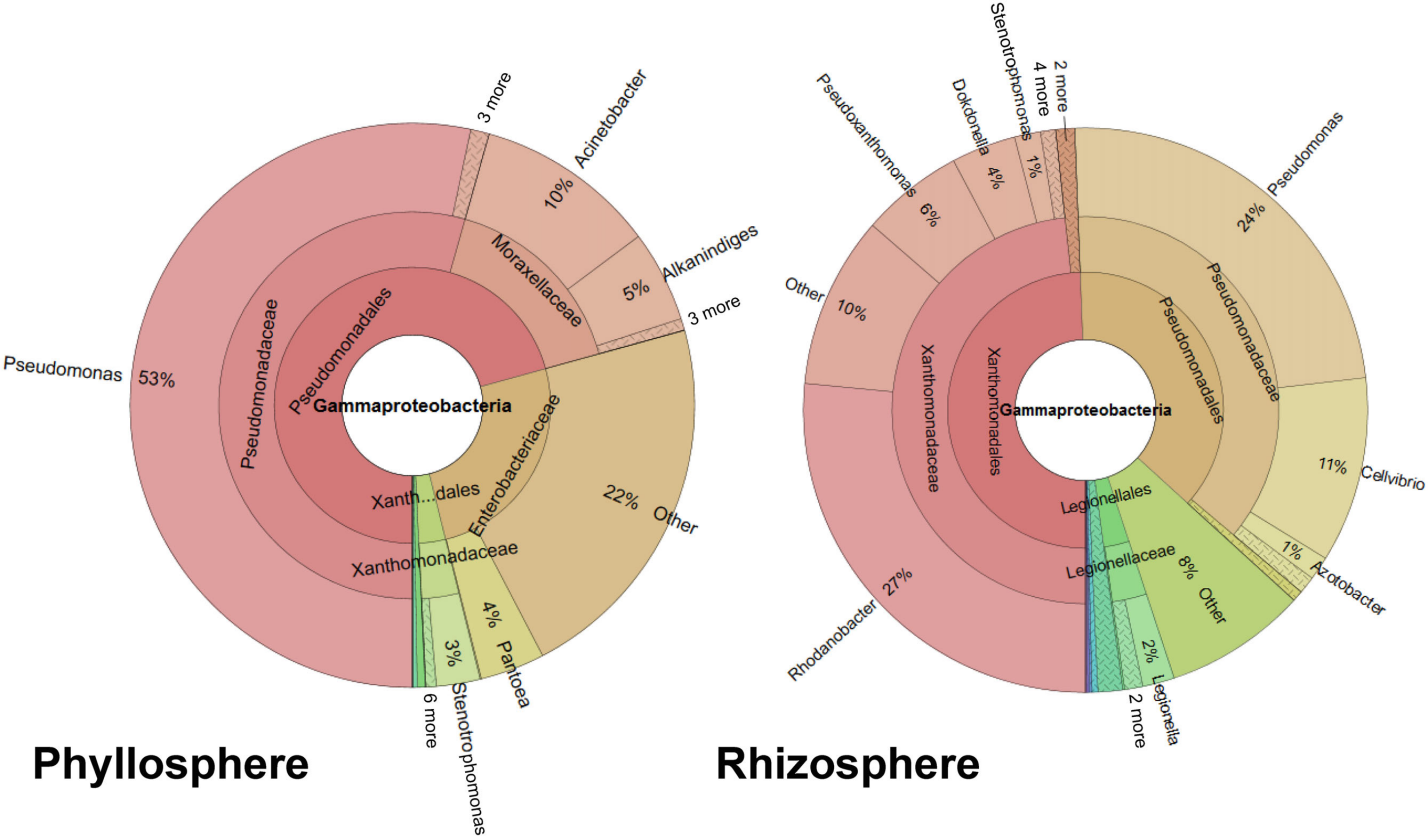
**Ring-charts showing the gammaproteobacterial community structure associated with investigated lettuce habitats**. The RDP classifier analysis is shown from the mean of 12 samples per habitat. The rings represent different taxonomic ranks (order, family, and genus), and the columns represent distinct taxa. Minor taxa are not specified.

Two sequential batches of 16S rRNA gene amplicon sequencing resulted in a total of 242,022 reads. After removing chimeras, singletons, and chloroplast sequences, 8233 quality mean reads per sample remained with a median absolute deviation of 1842.5 sequence reads. We analyzed the gammaproteobacterial fraction subjected to different treatments, separately for the phyllosphere and rhizosphere (Figure S1). Twenty-four samples (12 from per habitat) yielded in a total of 4.909 distinct OTUs, 1.102 were statistically different (Nonparametric ANOVA Kruskal Wallis Test, *p* ≤ 0.05) between both habitats. The gammaproteobacterial microbiome from whole lettuce plants contained mainly taxa from Pseudomonadales, followed by Xanthomonadales, Enterobacteriales and Legionellales (Figure 1). The rhizosphere was dominated by Xanthomonadaceae (48%) and Pseudomonadaceae (37%) while the dominating taxa associated to phyllosphere were Pseudomonadaceae (54%), Moraxellaceae (16%) and Enterobacteriaceae (25%). The genus *Pseudomonas* was almost exclusively assigned to the family Pseudomonadaceae (98%) associated to foliage. The most abundant genus of the root associated microbiome was *Rhodanobacter* (27%) followed by *Pseudomonas* (24%), while 8% of the rhizosphere associated reads could not be taxonomically assigned (Figure 1). In addition, differences were found between both plant development stages as well as between the different treatments (Figure S1). Interestingly, comparing only the phyllosphere samples of mature plants with bottom rot disease against the untreated control, we found 371 distinct OTUs with 99 statistically distinct differences.

### The core gammaproteobacterial community and its diversity

**Figure 2:**
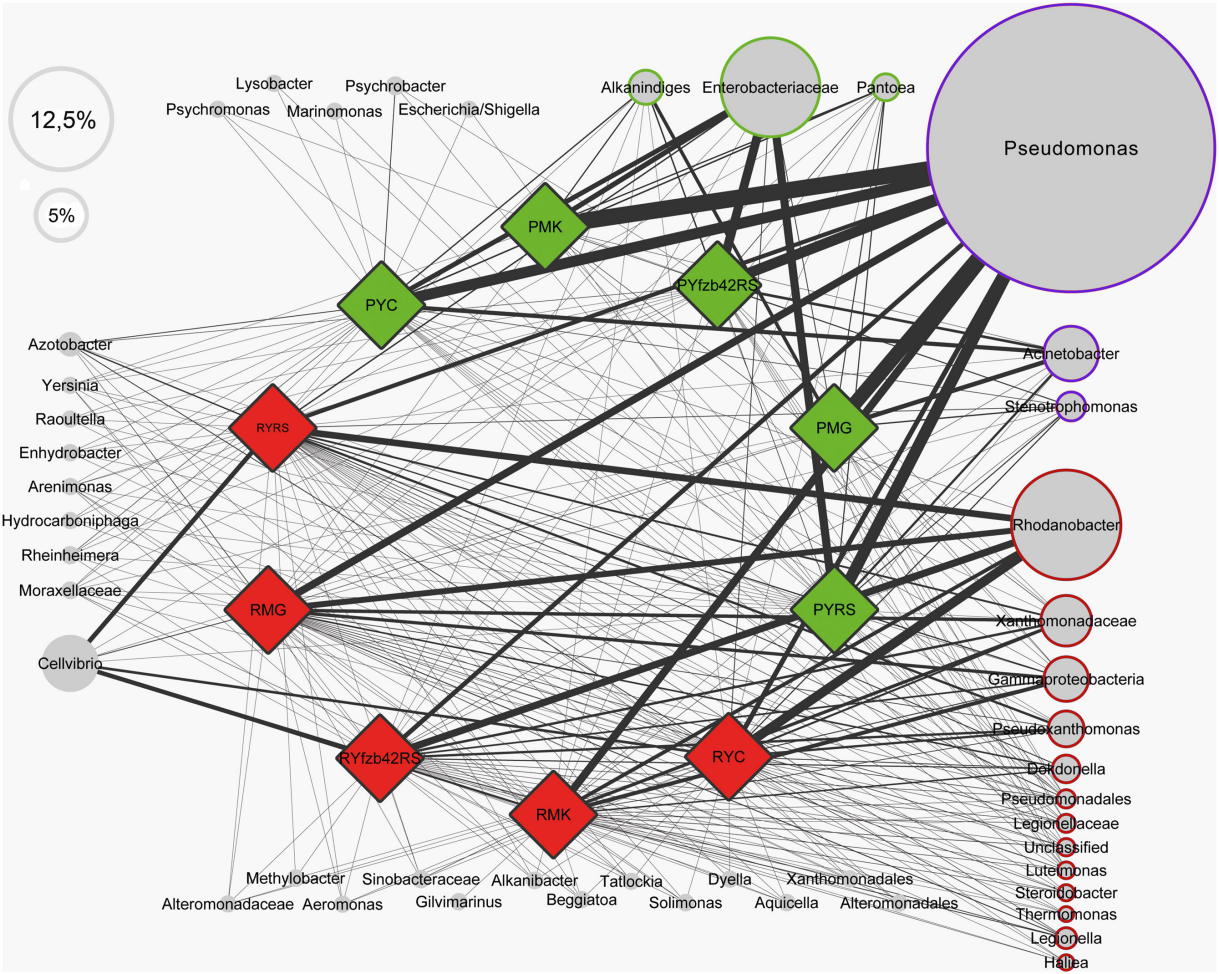
**Profile clustering network visualizing the investigated groups of lettuce (var. Tizian) and conjoined taxa**. Red diamonds indicate samples derived from the roots and green diamonds from the lettuce foliage, respectively. Nodes represent taxa derived from the RDP classifier and the node sizes correspond to the mean relative abundance of all samples. The abundance of a certain taxa correlated to a particular investigated group is visualized trough the line width of the corresponding connection. The frame color represents the affiliation to the observed core taxa (green—phyllosphere, red—rhizosphere, blue—occurrence in both habitats and all samples, white—not a core taxon). Taxa less abundant than mean ≤0.5% are displayed without size-correlation. Abbreviations: P, phyllosphere; R, rhizosphere; Y, young; M, mature; RS, *R. solani*; C, untreated (control); FZB42RS, FZB42 and *R. solani* co-inoculation; G, healthy; K, diseased.

A profile clustering network based on all 454-amplicon Gammaproteobacteria libraries was applied to visualize relationships of lettuce-associated bacterial genera and RDP-classified conjoined taxa. The network presented in Figure 2 can be used to identify the core microbiome of lettuce but also to connect specific taxa with different treatments, plant stages or with healthy and diseased plants. Our study revealed that plants belonging to different growth stages harbor several shared genera (at least 0.1% abundant in all samples compared). From 46 taxonomical assignments, 19 taxa could be assigned to a particular core microbiome structure of lettuce. Only *Alkanindiges*, *Pantoea*, and a lineage of Enterobacteriaceae (not classified at genus level) were exclusively assigned to the phyllosphere; they are indicated as phyllosphere core microbiome (Figure 2, surrounded by green color). For the rhizosphere a more diverse core microbiome was identified (red color): core taxa were assigned to *Rhodanobacter, Pseudoxanthomonas, Dokdonella, Luteimonas, Steroidobacter, Thermomonas, Legionella*, and *Haliea*. Additional OTUs were identified at family level as Xanthomonadaceae, Legionellaceae, Pseudomonadales, and Gammaproteobacteria. The highly abundant *Pseudomonas*, *Acinetobacter*, and *Stenotrophomonas* occurred in the core of both habitats (blue color). There are also other taxa found only associated to a specific habitat but did not account to any core group, due to low abundance or not comparable occurrence in the different treatments. In addition, detailed differences in the composition between the particular treatments are displayed by the network structure. For example, *Cellvibrio* was found preferentially in higher abundances in the rhizosphere of *R. solani* treated young plants and decreased in the rhizosphere of mature plants of the same treatment. Comparing all samples which were inoculated with *R. solani* (RS: PMK, PYRS, PYfzb42RS, *n* = 7) with the non-inoculated (C: PMG, PYC, *n* = 5), the highly abundant group of Enterobacteriaceae was dominantly retrieved from phyllosphere samples inoculated with *R. solani* (RS: 34.5% to C: 12.2% mean abundance), while Moraxellaceae including *Acinetobacter* and *Alkanindiges* were more abundant on the foliage of healthy plants (C: 30% to RS: 5.7% mean abundance). Similar gammaproteobacterial patterns could be observed on young plants inoculated only with *R. solani* compared to plantlets inoculated additionally with FZB42.

**Figure 3:**
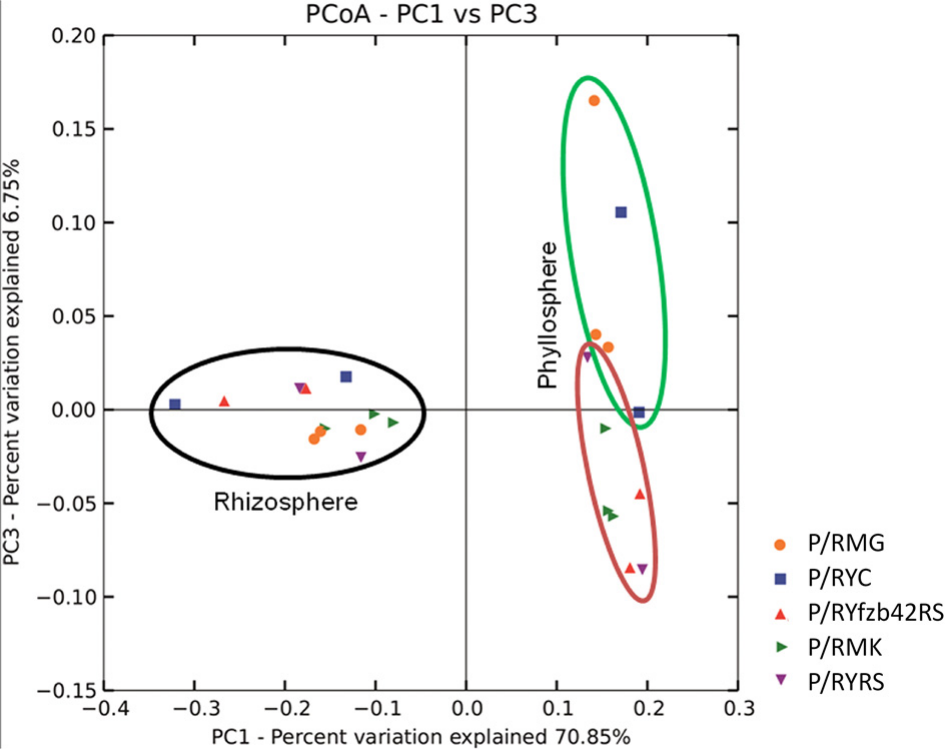
**Beta diversity metrics of bacterial 16S rRNA genes reveal distinctly clustered Gammaproteobacteria communities structured between healthy plants and plants affect by *Rhizoctonia solani***. Beta diversity community clustering is observed for phylogenetic beta diversity metrics (weighted UniFrac). In the panel, each point corresponds to a sample from either the lettuce rhizosphere (black) or the phyllosphere (green and red). Red—samples inoculated with *R. solani;* green—untreated control group. The percentage of variation explained by the plotted principal coordinates is indicated on the axes.

**Figure 4:**
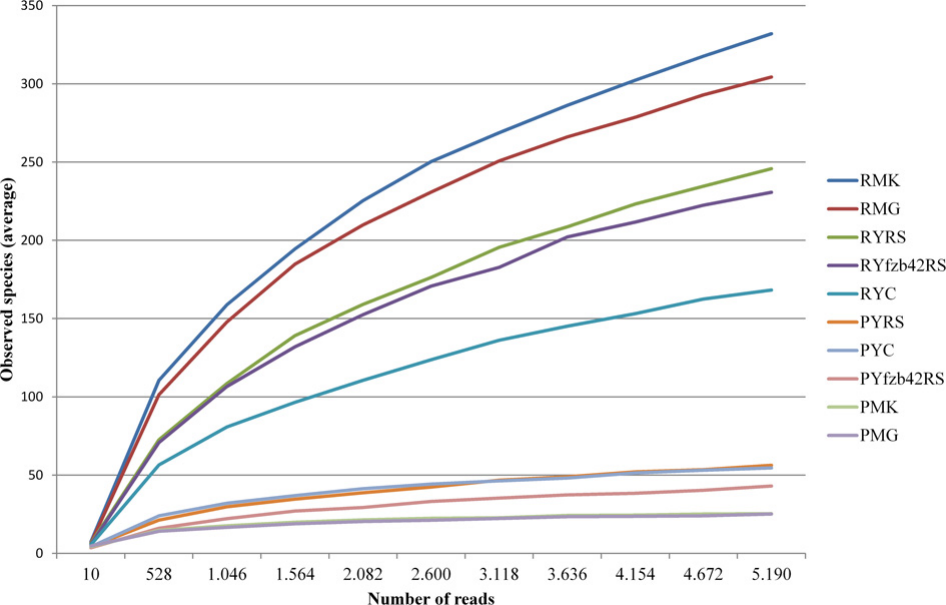
**Rarefaction analysis comparing overall diversity of the indigenous microbiota of the investigated lettuce samples (var. Tizian)**. Prior to rarefaction analysis, rhizosphere, and phyllosphere libraries were pairwise combined corresponding to the particular treatments. Rarefaction curves show saturation of the combined datasets that were clustered at 97% sequence similarity. The curves are supported by 95% confidence intervals. The overall diversity was higher affected in the rhizosphere. In both habitats plants treated with *Rhizoctonia solani* showed higher diversity than plants treated additionally with FZB42 or untreated plants. Abbreviations: P, phyllosphere; R, rhizosphere; Y, young; M, mature; RS, *R. solani*; C, untreated (control); FZB42RS, FZB42 and *R. solani* co-inoculation; G, healthy; K, diseased.

Beta diversity (pairwise sample dissimilarity) indices based on weighted UniFrac distances revealed clear differences between the habitats, but also between young and mature plants infected by *R. solani* with bottom rot disease compared to non-inoculated healthy young and mature plants (Figure 3, Table S1). A general higher variation was observed among samples derived from the phyllosphere. Alpha diversity indices (Table S1) based on the observed species metric showed a higher diversity of Gammaproteobacteria associated with plants inoculated with *R. solani*, but the co-inoculation with FZB42 seems to reduce this effect (Figure 4). This was observed across both investigated habitats, and additionally a slight increase of the overall diversity correlated with the maturity state.

## Discussion

Our study gave new insights into the general structure of the lettuce microbiome as well as showed the impact of the plant pathogen *R. solani* AG1-IB and its antagonistic counterpart *B. amyloliquefaciens* FZB42. Sequencing of 16S rRNA gene amplicons provided especially a deeper look into the fraction of often health relevant Gammaproteobacteria in the lettuce-associated microbiome, down to the taxonomic rank of genera. While *R. solani* is a serious lettuce pathogen (Wolf and Verreet, 1999), Chowdhury et al. (2013) demonstrated that FZB42 is an efficient biocontrol agent. FZB42 was able to effectively reduce the disease severity of bottom rot caused by *R. solani* in pot and field experiments. In our study we showed that both microorganisms have not only a significant impact on plant health, they also significantly influence the structure of the plant-associated microbiome.

The rhizosphere and phyllosphere gammaproteobacterial microbiomes of healthy lettuce plants showed significant differences in their overall composition, their core, and diversity indices. This can be explained by the completely different abiotic conditions in both plant habitats (Raaijmakers et al., 2009; Vorholt, 2012). Here, the most significant and surprising difference we found was the preferential occurrence of enterics in the phyllosphere. Our results are in accordance with the principal findings of Rastogi et al. (2012), who analyzed spatiotemporal variation in bacterial community composition on field-grown lettuce in California. The general composition of phyllosphere bacteria was similar, and Enterobacteriacaea were a substantial fraction also in this study. In general, plant microhabitats are a reservoir for Enterobacteriaceae including potentially human pathogenic bacteria such as human enteric pathogens (Brandl, 2006). Especially after intermediate disturbances such as plant diseases, their abundance was enhanced (Erlacher et al., unpublished data). Due to their impact on human health as either pathogens or immunostimulants, this is an observation that could be of considerable importance for health concerns. Hanski et al. (2012) could show correlation between bacterial diversity and atopy, suggesting significant interactions with Gammaproteobacteria. These authors further showed a positive association between the abundance of *Acinetobacter*, found abundantly in healthy lettuce in our study and Interleukin-10 expression in peripheral blood mononuclear cells in healthy human individuals. Interleukin-10 is an anti-inflammatory cytokine and plays a central role in maintaining immunologic tolerance to harmless substances (Eskdale et al., 1997; Lloyd and Hawrylowicz, 2009).

In general, members of the genus *Pseudomonas* play a prominent role in the lettuce gammaproteobacterial microbiome. They were the dominant inhabitants of the phyllosphere; at family level pseudomonads present 54% and at genus level 53% of the microbiome. Also in the rhizosphere they represent 37% of the OTUs at family level. Altogether, *Pseudomonas* was the most dominant member of the lettuce core microbiome. *Pseudomonas* is a model organism to study beneficial plant-microbe interactions (Haas and Défago, 2005). Interestingly, in our study, *Pseudomonas* was not only related to healthy plants, there was also strong connection to diseased plants. The fact is not surprising because pathogenic pseudomonads are well-known but shows the limitation of the applied method. Using amplicon sequencing we can identify the genus or species but not their functional traits. For functional analysis metagenomic/transcriptomic techniques are required.

Plant-microbe interactions are highly complex and changes in the abundance of individual strains, either pathogens or beneficials can result in non-linear alterations of the entire microbiome composition. Such alterations may lead to negative effects to plants and humans as consumers (Berg et al., 2005). However, microbiome shifts can hardly be predicted and must be traced by thorough screening using culture-independent and sequencing-based approaches. Adesina et al. (2009) used DGGE fingerprint to study microbiome shifts and showed that *R. solani* AG1-IB inoculation severely affected the bacterial and fungal community structure in the rhizosphere of lettuce and that these effects were much less pronounced in the presence of the antagonistic counterpart *P. jessenii* RU47. In our study we used amplicon sequencing, which allowed a deep insight into the composition of Gammaproteobacteria. The human health relevant group of Enterobacteriaceae was affected by the *R. solani*; we found a significant increase in gammaproteobacterial diversity due to the pathogen outbreak. The overall enhancement of diversity after biotic stimulation by a pathogen agrees well with the intermediate disturbance hypothesis known to ecology from studies of higher plants or coral reefs (Connell, 1978). However, together with FZB42 this increase was less distinct. Until today, the mechanisms described for biocontrol agents focus on direct antagonistic effects against a pathogen or an interaction via the plant's immune system (Doornbos et al., 2012). In this study we showed a selective compensation of the impact of a pathogen on the indigenous plant-associated microbiome by the biocontrol agent, which is an interesting effect of the beneficial aspect of the inoculant.

Biocontrol of plant pathogens is a promising solution for sustainable agriculture. Molecular techniques, which allowed a deeper insight into the crop-associated microbiome, can also be applied to develop new biocontrol strategies (Berg et al., 2013). Using a profile clustering network in our study, *Acinetobacter* and *Alkanindiges* were identified as indicators of healthy lettuce plants. Therefore, they could be promising biocontrol agents. An endophytic *Acinetobacter* strain isolated from healthy stems of the plant *Cinnamomum camphora* was already used as biocontrol strain against fungal diseases (Liu et al., 2007) but nothing is known about any biocontrol activity of *Alkanindiges*. In contrast, in the study of Rastogi et al. (2012), the foliar presence of *Xanthomonas campestris* pv. vitians, which is the causal agent of bacterial leaf spot of lettuce, correlated positively with the relative representation of bacteria from the genus *Alkanindiges*. Here, more research is needed for understanding bacterial networking on plants, which is an essential step toward predictable biocontrol. In addition, beneficial *Pseudomonas* strains could be other interesting candidates for biocontrol because we found a strong connection to healthy lettuce plants. This was shown already successfully for *P. jessenii* RU47 by Adesina et al. (2009). Altogether, new results favorite diverse bacterial cocktails to control plant diseases (Berg et al., 2013); for lettuce they could contain *Acinetobacter, Bacillus*, *Pseudomonas*, and *Serratia* strains as well.

### Conflict of interest statement

The authors declare that the research was conducted in the absence of any commercial or financial relationships that could be construed as a potential conflict of interest.
