# Supplementary material for: The impact of the pathogen Rhizoctonia solani and its beneficial counterpart Bacillus amyloliquefaciens on the indigenous lettuce microbiome
Source: Front Microbiol. 2014 Apr 21;5:175. doi: 10.3389/fmicb.2014.00175 (PMC4001036; doi:10.3389/fmicb.2014.00175)

**SUPPORTING MATERIAL**

**The impact of the pathogen *Rhizoctonia solani* and its beneficial counterpart *Bacillus amyloliquefaciens* on the indigenous lettuce microbiome**

Armin Erlacher^1,2^, Massimiliano Cardinale^1,2^, Rita Grosch^3^, Martin Grube^2^, and Gabriele Berg^1^

^1^Institute of Environmental Biotechnology, Graz University of Technology, Petersgasse 12, 8010 Graz, Austria

^2^Institute of Plant Sciences, University of Graz, Holteigasse 6, 8010 Graz, Austria

^3^Institute of Vegetable and Ornamental Crops, Grossbeeren, Germany

*Corresponding Author, E-mail: gabriele.berg@tugraz.at

Tel: +43-316-873-8310

Table S1 Richness estimates and diversity indices obtained.

| **Treatment** | **shannon** | **equitability** | **dominance** | **simpson** | **simpson_reciprocal** | **simpson_e** | **PD_whole_tree** | **chao1** | **observed_species** |
| --- | --- | --- | --- | --- | --- | --- | --- | --- | --- |
| PMK 1 | 2.47 | 0.51 | 0.24 | 0.76 | 4.17 | 0.14 | 2.46 | 31.00 | 29 |
| PMK 2 | 1.81 | 0.38 | 0.46 | 0.54 | 2.18 | 0.08 | 1.99 | 29.00 | 28 |
| PMK 3 | 2.57 | 0.57 | 0.22 | 0.78 | 4.63 | 0.20 | 1.94 | 26.75 | 23 |
| PMG 1 | 1.83 | 0.38 | 0.45 | 0.55 | 2.22 | 0.08 | 3.03 | 29.50 | 27 |
| PMG 2 | 2.77 | 0.55 | 0.22 | 0.78 | 4.54 | 0.14 | 3.22 | 50.33 | 32 |
| PMG 3 | 2.93 | 0.60 | 0.17 | 0.83 | 5.72 | 0.19 | 3.62 | 37.50 | 30 |
| PYfzb42RS 1 | 1.94 | 0.37 | 0.33 | 0.67 | 3.06 | 0.08 | 3.56 | 42.00 | 37 |
| PYfzb42RS 2 | 2.17 | 0.34 | 0.35 | 0.65 | 2.85 | 0.04 | 4.96 | 81.00 | 81 |
| PYRS 1 | 2.37 | 0.38 | 0.32 | 0.68 | 3.11 | 0.04 | 5.35 | 96.10 | 73 |
| PYRS 2 | 2.42 | 0.39 | 0.31 | 0.69 | 3.17 | 0.04 | 5.35 | 103.08 | 74 |
| PYC 1 | 1.78 | 0.33 | 0.55 | 0.45 | 1.82 | 0.04 | 4.07 | 45.50 | 42 |
| PYC 2 | 3.26 | 0.50 | 0.16 | 0.84 | 6.28 | 0.07 | 5.92 | 133.86 | 91 |
| RMK 1 | 5.40 | 0.64 | 0.08 | 0.92 | 12.59 | 0.03 | 24.60 | 570.80 | 362 |
| RMK 2 | 5.01 | 0.59 | 0.13 | 0.87 | 7.89 | 0.02 | 23.72 | 495.05 | 362 |
| RMK 3 | 5.32 | 0.63 | 0.08 | 0.92 | 13.17 | 0.04 | 23.04 | 545.59 | 361 |
| RMG 1 | 4.68 | 0.56 | 0.14 | 0.86 | 7.00 | 0.02 | 22.29 | 456.27 | 328 |
| RMG 2 | 4.96 | 0.58 | 0.12 | 0.88 | 8.61 | 0.02 | 24.76 | 644.15 | 369 |
| RMG 3 | 4.63 | 0.56 | 0.13 | 0.87 | 7.86 | 0.03 | 19.24 | 391.02 | 296 |
| RYfzb42RS 1 | 4.07 | 0.48 | 0.17 | 0.83 | 5.84 | 0.02 | 21.96 | 511.02 | 351 |
| RYfzb42RS 2 | 3.94 | 0.48 | 0.14 | 0.86 | 6.98 | 0.02 | 17.75 | 379.54 | 290 |
| RYRS 1 | 4.03 | 0.48 | 0.16 | 0.84 | 6.31 | 0.02 | 21.33 | 430.40 | 340 |
| RYRS 2 | 4.29 | 0.53 | 0.11 | 0.89 | 9.04 | 0.03 | 18.17 | 399.12 | 263 |
| RYC 1 | 4.02 | 0.50 | 0.16 | 0.84 | 6.25 | 0.02 | 17.58 | 397.52 | 270 |
| RYC 2 | 2.71 | 0.37 | 0.40 | 0.60 | 2.52 | 0.02 | 10.26 | 220.63 | 161 |

Abbreviations: P – phyllosphere. R – rhizosphere; Y – young. M – mature; RS – *R. solani*. C – untreated (control). FZB42RS – FZB42 and *R. solani* co-inoculation; G – healthy. K – diseased

Figure S1 Heatmap based on the relative abundance of obtained OTUs derived from all samples. Only groups ≥200 counts per OTU are displayed. The vertical columns represent the investigated samples. The horizontal axes depict OTUs with the taxonomic affiliation. A color change from cold to warm colors indicates an abundance increase.


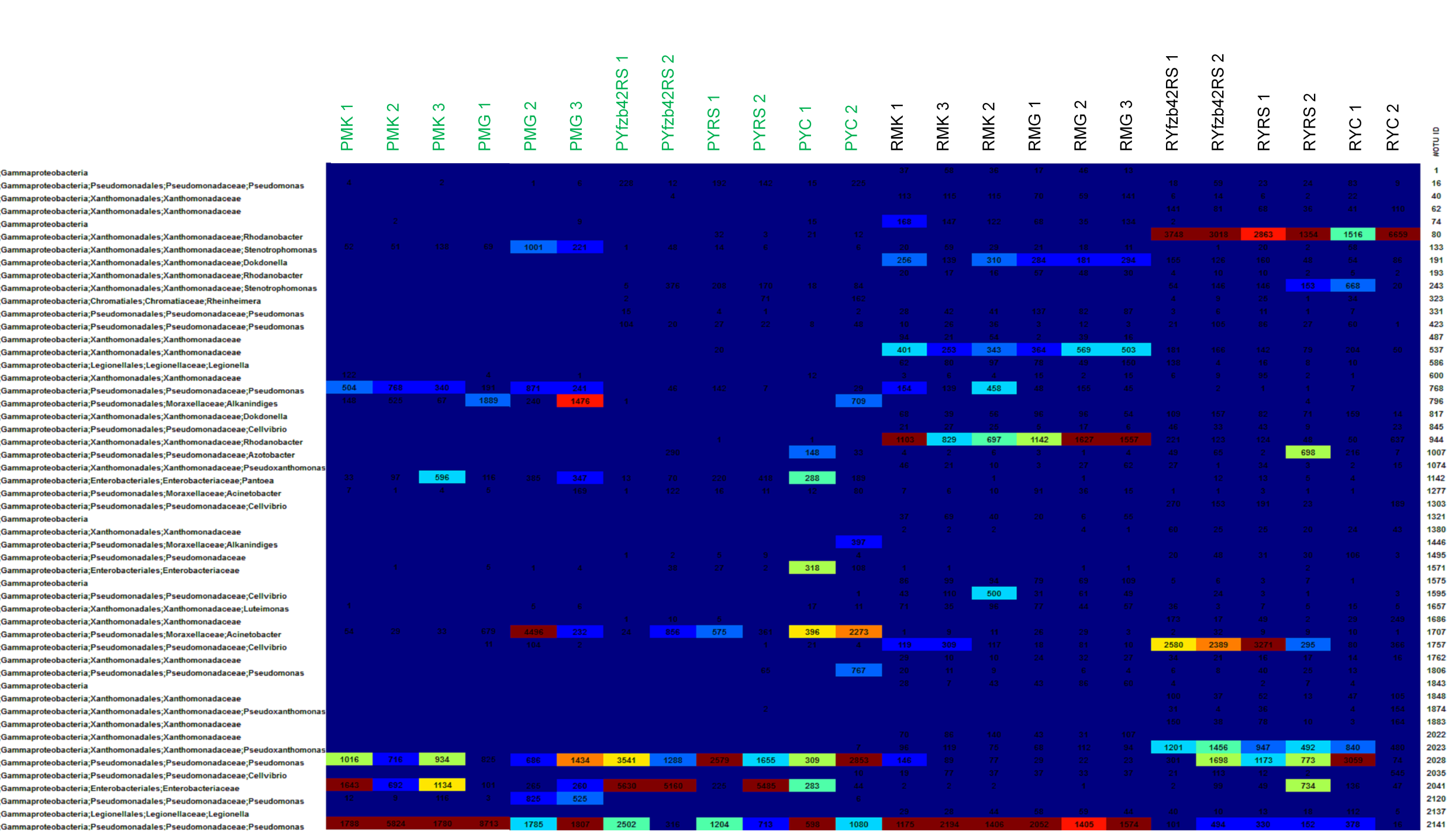

Supplement: Supplementary file 1 [file DataSheet1.DOCX]
